# Coastal flooding and mean sea-level rise allowances in atoll island

**DOI:** 10.1038/s41598-022-05329-1

**Published:** 2022-01-24

**Authors:** Angel Amores, Marta Marcos, Gonéri Le Cozannet, Jochen Hinkel

**Affiliations:** 1 Instituto Mediterráneo de Estudios Avanzados (UIB-CSIC), Esporles, Spain; 2 Departament de Física (UIB), Palma, Spain; 3 French Geological Survey (BRGM), Orléans, France; 4 Global Climate Forum (GCF), Berlin, Germany

**Keywords:** Climate-change impacts, Natural hazards, Climate-change mitigation

## Abstract

Atoll islands are among the places most vulnerable to climate change due to their low elevation above mean sea level. Even today, some of these islands suffer from severe flooding generated by wind-waves, that will be exacerbated with mean sea-level rise. Wave-induced flooding is a complex physical process that requires computationally-expensive numerical models to be reliably estimated, thus limiting its application to single island case studies. Here we present a new model-based parameterisation for wave setup and a set of numerical simulations for the wave-induced flooding in coral reef islands as a function of their morphology, the Manning friction coefficient, wave characteristics and projected mean sea level that can be used for rapid, broad scale (e.g. entire atoll island nations) flood risk assessments. We apply this new approach to the Maldives to compute the increase in wave hazard due to mean sea-level rise, as well as the change in island elevation or coastal protection required to keep wave-induced flooding constant. While future flooding in the Maldives is projected to increase drastically due to sea-level rise, we show that similar impacts in nearby islands can occur decades apart depending on the exposure to waves and the topobathymetry of each island. Such assessment can be useful to determine on which islands adaptation is most urgently needed.

## Introduction

Atoll islands are arguably the places most vulnerable to climate change because they consist of small areas of land that are only 1–5 m above mean sea levels^1–5^. Already today, this makes atolls vulnerable to flooding from waves, a natural process that originated these islands in the past and sustains them but that currently also affects human populations and infrastructures. Waves caused either by tropical cyclones (e.g.^6^) or remotely-generated swells (e.g.^7–9^), which can reach significant wave heights of, at least, 5 m^10^, have the potential to cause damages to buildings, infrastructure, agricultural production, freshwater lenses and to even threaten human safety due to the lack of sheltered elevated areas. Climate change and mean sea-level rise exacerbate this vulnerability by increasing extreme water levels.

Against this backdrop, it has been conjectured that most atolls could become uninhabitable during the 21st century, especially those that lack the financial resources for large-scale and costly adaptation measures^3^. This conjecture, however, was based on a study of a single island (Roi-Namur Island on Kwajalein Atoll in the Republic of the Marshall Islands) while atoll islands are diverse both in terms of topobathymetric setting (e.g. reef width)^10^ and exposure to waves^9^. Furthermore humans living on atoll islands are adapting these islands either by artificial infrastructures (breakwaters, dikes, etc.), elevating buildings and raising whole islands^2,11–13^, or by reinstalling ecosystem services such as the natural capacity of islands to accrete vertically with mean sea-level rise^2,14,15^ provided sufficient sediment is available and the time span is long enough^16^.

Hence, from a policy coastal-management perspective the crucial questions that need to be addressed are: (1) for how long a given atoll island is safe from mean sea-level rise and associated wave-induced flooding if no adaptation action is taken (that is, no increases in flooding hazards are expected), and (2) by how much do flood defenses or island elevations need to be raised in order to keep islands safe until a given moment in time (as mean sea level rises)?

Importantly, these questions need to be addressed simultaneously for many of rapidly artificially developing atoll islands belonging to small island states^12,13,17^, in order to advance regional development and infrastructure planning in face of relative sea-level rise, which is a priority in such dispersed island nations^12,18,19^. The dilemma thereby is that wave interaction with atoll coral reefs is a complex physical process that involves strong wave dissipation, generation of infragravity waves over the reef flat, and wave setup and run-up at the shoreline, that ultimately may lead to overtopping and overflow on the island^20^. All these effects were thought to be only captured accurately by computationally demanding wave models, which is why previous works have focused on single island case studies^3,12,21^.

**Figure 1:**
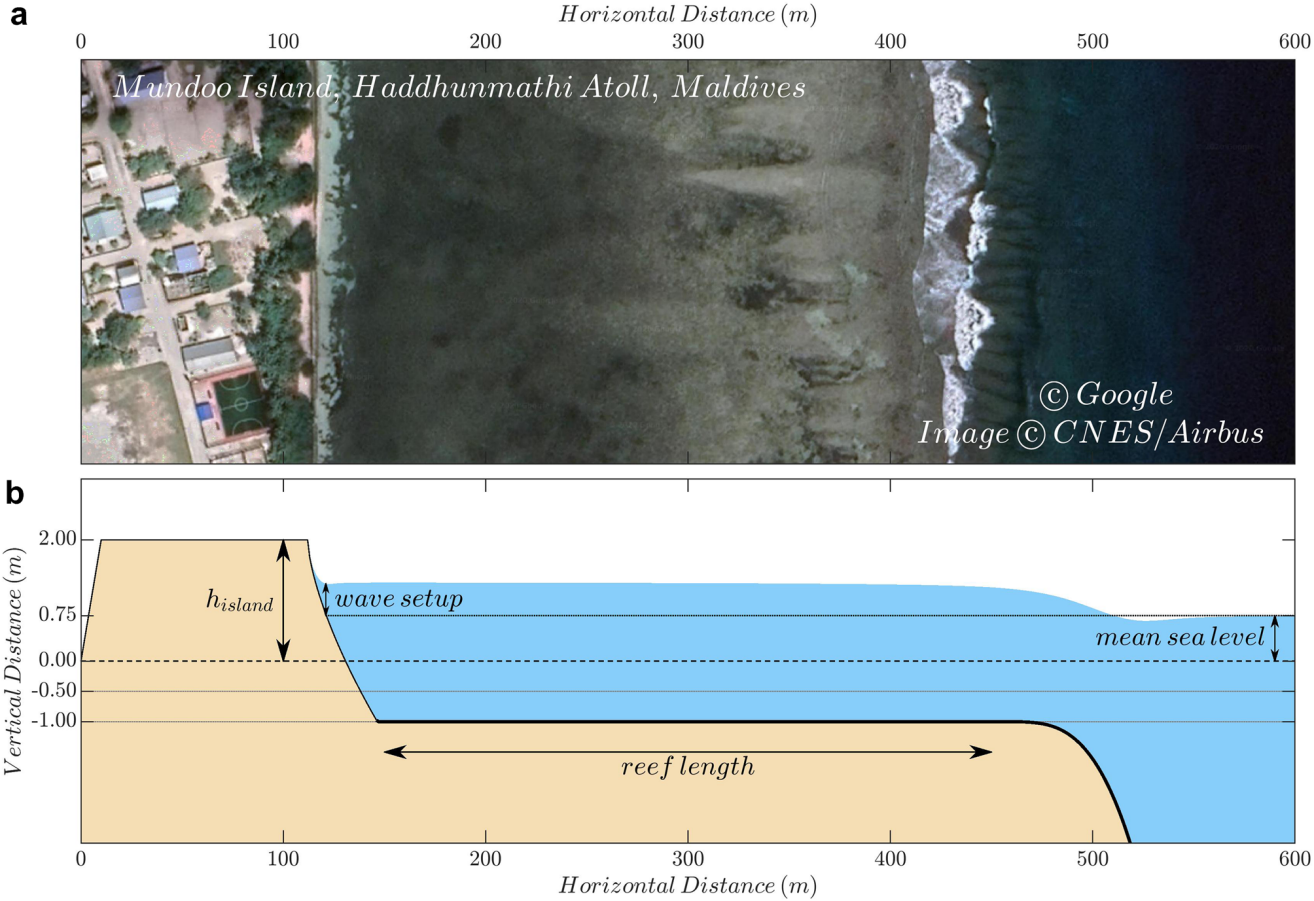
(**a**) Image of the coast of Mundoo Island ($73.535230^{\circ }$ E, $2.012024^{\circ }$ N), located at Haddhunmathi Atoll, Maldives, extracted from Google Earth and processed with Matlab. (**b**) Example of a profile used in a simulation with the resulting wave setup for a $H_s$ of 3 m, $T_p$ of 15 s, island height of 2 m, 0.75 m of mean sea level, 300 m of reef length and 0.1 of Manning’s friction coefficient over the reef. The thin (thick) black line over the topobathymetric profile indicates that a 0.025 (0.1) value of the friction coefficient has been used for the beach (reef). The wave setup for the subsequent parameterisation was extracted at sea level point on the beach, as indicated in the figure. The flooding was computed as the total water that reached the point (0,0) of the profile.

Here we present a large set of numerical simulations that provide the wave-induced flooding over an atoll island as a function of the incoming wave parameters (namely significant wave height, $H_s$, and peak period, $T_p$), the Manning’s friction coefficient, the length of the reef flat and the island height, under different mean sea level conditions. These simulations are furthermore used to derive a simple parameterisation of the wave setup at the shoreline of the atoll island that can be easily applied. A similar parameterisation approach, but adapted to other coastline types, has been used successfully in other world regions, as for example EurOTop (http://www.overtopping-manual.com/) that provides wave-induced flooding in common coastal configurations in Europe based on physical model tests. The wave setup parameterisation and the estimation of the wave-induced flooding have been derived using more than 194,000 numerical simulations with a non-hydrostatic, phase-resolving wave model^22^ under varying conditions of wave forcings and mean sea levels (with values up to 1.25 m above present-day), for reef profiles (Fig. 1) that are typical of reef islands in the Indian and Pacific Oceans^23^, with reef lengths and land heights up to 600 m and 2.5 m, respectively. The Manning’s friction coefficients of the reef slope and reef flat range from 0.025 up to 0.2 (see “Methods”).

As we find a strong correspondence between parameterised and modelled wave setup, we anticipate that our parameterisation and the set of simulations will be able to support the identification of the most vulnerable locations to flooding and broad-scale coastal planning in reef environments, saving large computational efforts. We illustrate the usefulness of our approach with a case study for 56 atoll islands in the Maldives for which elevations and reef lengths are known from land surveys and satellite images. Using the simulations we estimate under what value of increased mean sea level and its associated time frame, each of these islands will cross a user-defined threshold of tolerable annual flooding and hence may be considered ‘unsafe’. We also calculate by how much the islands need to be raised to cope with higher mean sea levels. To do so, we use three different approaches: (1) raising the islands the same amount as the mean sea-level rise, (2) keeping the total water level constant (defined here as mean sea level plus wave setup) or (3) keeping the probability of flooding constant under mean sea-level rise, and compare their outputs in terms of flooding.

## Results

### Parameterisation of wave-setup and computation of the wave-induced flooding

Results indicate that wave setup depends linearly on the wave forcing and decreases with higher mean sea levels, a behaviour already proved in laboratory experiments^24^. No correspondence was found with the island height (Figure S2, see “Methods”), as the island elevation does not play a role unless there is overflow. Furthermore, the reef length and Manning’s friction coefficient only influence the transitory state but neither the stationary wave regime nor the wave setup over the submerged reef flat. Note that the time to reach the steady wave setup does depend on the reef length and Manning’s friction coefficient due to the longer time needed to accumulate the water near the shoreline.

**Figure 2:**
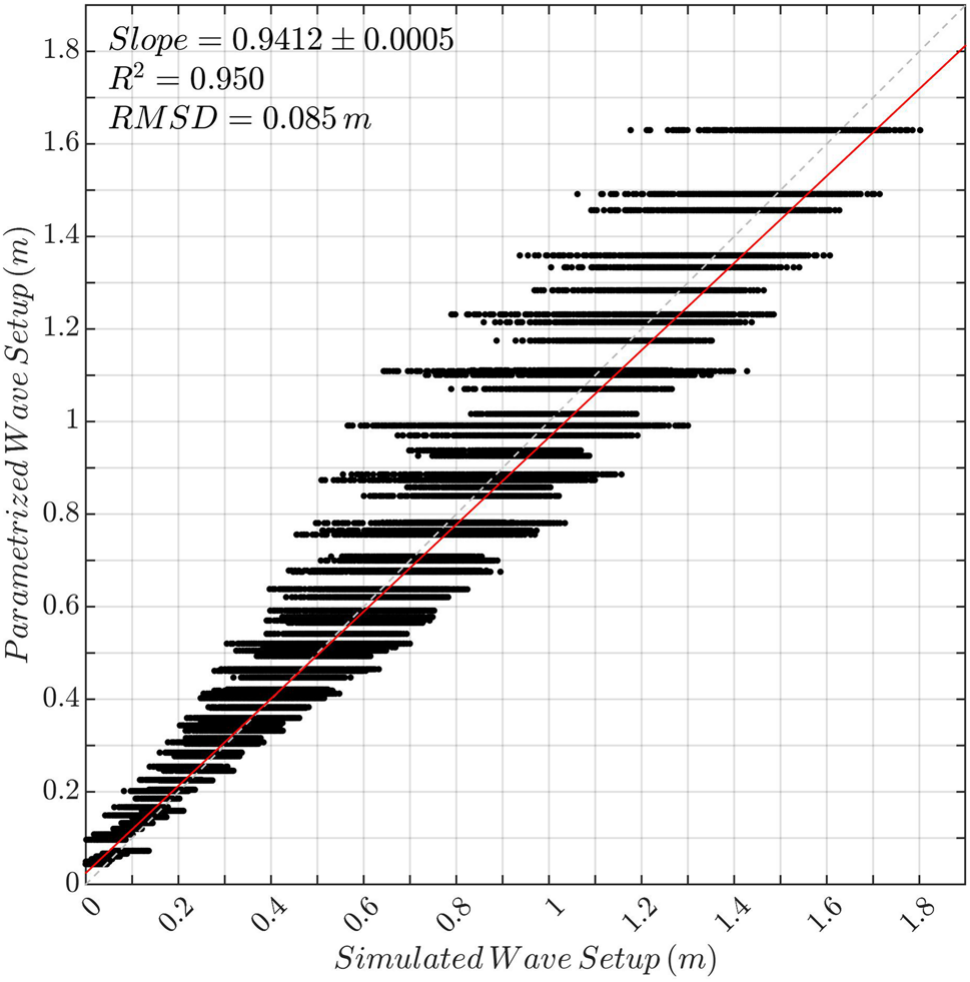
Parameterised wave setup computed using Eq. (1) vs modelled wave setup.

Based on the modelled relationship for the wave setup described above, a parameterisation has been derived and has been fitted to the numerical model outputs through non-linear-least-squares (see “Methods” for details). The comparison between modelled and parameterised values of all variables involved, shown in Fig. 2, demonstrates an excellent agreement, with overall $R^2$ greater than 0.95 and a root-mean-square-difference of 8.5 cm. This close correspondence, valid for a wide range of island reef morphologies and input waves, implies that wave setup along reef islands can be estimated using our parameterisation, instead of a computationally-intensive numerical model.

Unlike in wave setup, wave-induced flooding is dependent upon both incoming wave characteristics and island morphology. Flooding increases with the wave forcing and mean sea-level, and decreases with the island height, reef length and friction coefficient. While it is obvious that higher island elevations prevent flooding, the decrease in flooding with increasing friction coefficient and reef lengths is explained by the higher wave energy dissipation. As the wave-induced flooding is dependent upon all the tested parameters, there is no simple parameterisation that can be used as surrogate model in this case. Therefore, the approach followed for the wave setup is not appropriate and the model outputs need to be used directly, thus increasing computational demand. The results of the 194,000 simulations have been directly applied to compute changes in the flooding as mean sea level rises (see “Methods” and Fig. S4 for further details).

### Application to the Maldives

The potential of these results for a broad scale assessment of flooding hazards in coral reef environments is illustrated for a case study in the Maldives under a set of state-of-the-art mean sea-level rise scenarios and regional wave climate data.

**Figure 3:**
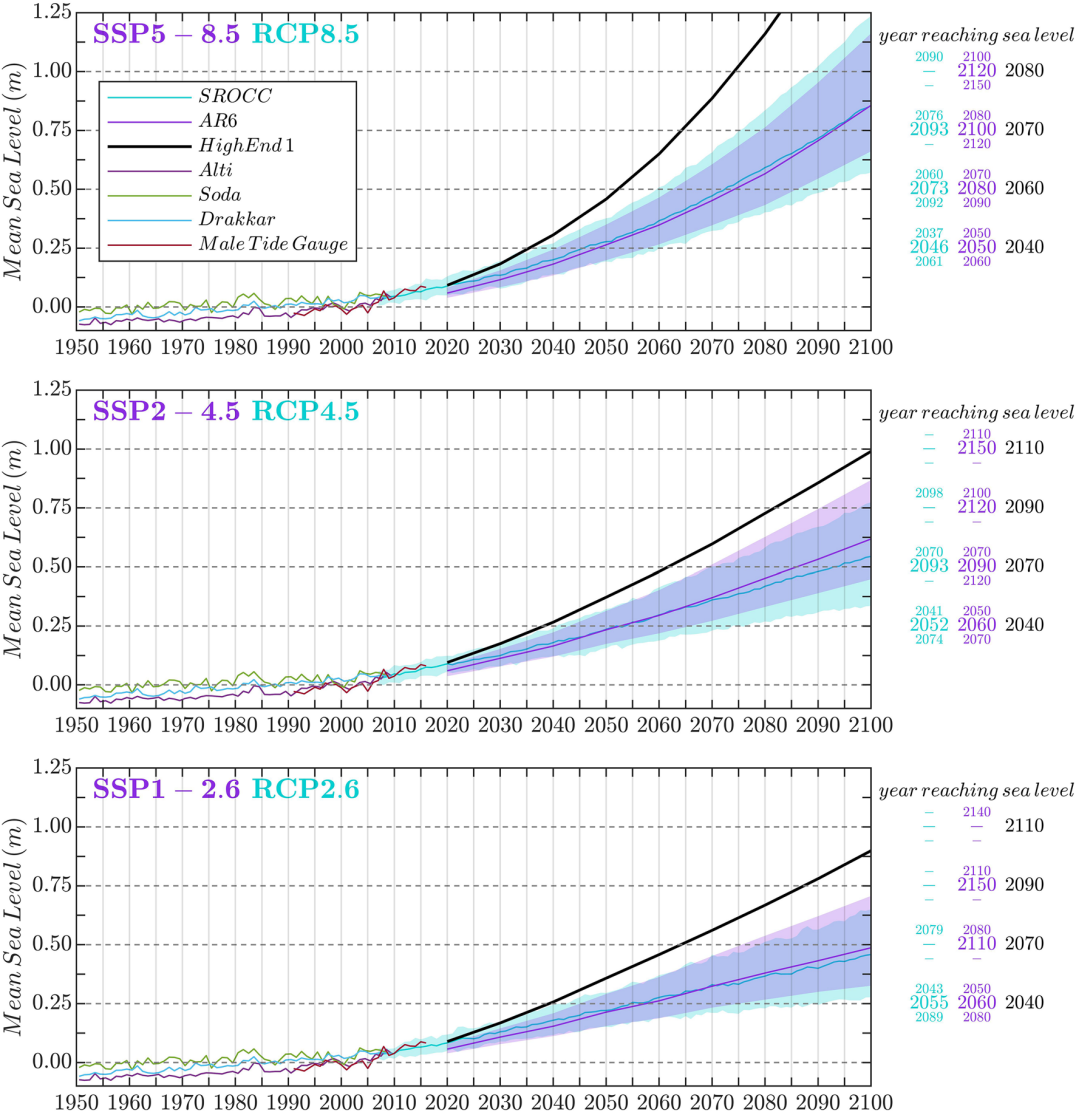
Mean sea-level reconstructions from observations (1950–2000) and likely projections, median and high-end scenarios for the Maldives (Malé) during the 21st century (based on data and methods of^18,28,56,58,59^), with the associated periods of time by which elevations of 0.25, 0.5, 0.75 and 1 m are projected to be exceeded, with respect to the average mean sea level of 1986–2005.

The Maldives consist of 1192 coral reef islands dispersed across 860 km in the tropical Indian Ocean and inhabited by more than half a million people (2020). With land elevations ranging between 0.5 and 2.3 m above present-day mean sea level^25,26^ and most of the islands being lower than 1 m, the Maldives is among the countries with lowest average land elevations in the world and, hence, among the places most vulnerable to the increased frequency of extreme sea levels caused by mean sea-level rise induced by climate change^7,12^. Various 21st century relative sea-level rise projections for the Maldives are shown in Fig. 3 for three Shared Socio-Economic Pathways (SSP) and Representative Concentration Pathways (RCP). Extreme sea levels can potentially flood islands completely, as it happened on 15–17 May 2007, when multiple consecutive swell events generated at the Southern Ocean hit the archipelago^7^, causing substantial erosion, damaging harbours, quay walls and houses, and affecting basic services such as electricity, water and sewerage systems^27^. This kind of events will become more likely with higher mean sea levels because waves will reach the islands coastlines upon higher total water levels. As a result, in absence of adaptation, Maldivian islands (and presumably all atoll nations) are threatened to become uninhabitable much before being completely submerged by rising mean sea level.

**Figure 4:**
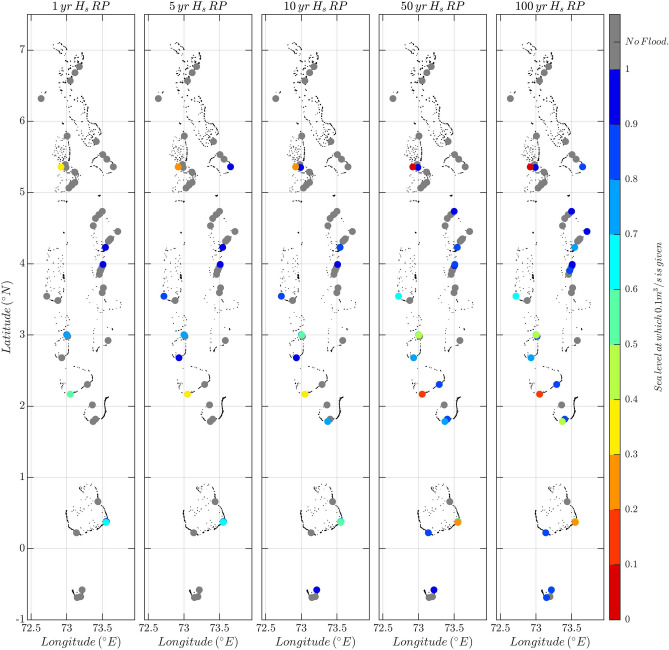
Mean sea-level rise required (analysed up to 1 m) to generate a flooding above 0.1 m$^3$/s for different return periods of wave heights. The wave-induced flooding derived from the simulations is applied to 56 Maldivian atoll islands where elevation and reef lengths are known (dots) under different return periods of $H_s$ between 1 and 100 years with a Manning friction coefficient of 0.1. The coloured dots indicate at which mean sea level a flooding larger than 0.1 m$^3$/s is given. The grey dots are islands that do not get flooded with a mean sea-level rise up to 1 m.

### How long are Maldivian islands habitable?

To answer the first policy question until when a given atoll island remains safe from mean sea-level rise and associated wave-induced flooding assuming no topobathymetric change, we have applied the results of the flooding simulations to the 56 Maldivian islands for which sufficient morphology information is available. The values of $H_s$ and $T_p$ of the incoming waves are different for every island and are derived from^9^ (see “Methods”). Figure 4 shows the location of these islands (dots); it also shows at what mean sea level above the present-day value each island will be flooded (with a predefined *critical flooding threshold* greater than 0.1 m$^3$/s per linear meter of coastline) under a wave forcing corresponding to return periods between 1 and 100 years (note that these values are site-dependent). The Manning friction coefficient has been fixed to 0.1 (the same results are shown in Fig. S5 for a smaller Manning coefficient of 0.05). Note that the flooding threshold is illustrative and in practice needs to reflect the risk preference of the stakeholders affected. Here, we have taken this value from the French standards (http://infoterre.brgm.fr/rapports/RP-64807-FR.pdf)^9^, which associate the value of 0.1 m$^3$/s per linear meter to moderate risk for human life. With the given set of parameters (flooding threshold and Manning coefficient) we find, for example, that a total of two islands, out of 56, experience flooding above the defined threshold at least once every 5 years when mean sea level reaches 0.5 m (see the yellow and orange dots in the second panel in Fig. 4). Under RCP8.5, this occurs in year 2073 (likely range 2060–2092) according to the projections of^18^ and in 2052 according to the high end scenario of^28^ (see Fig. 3). It must be remarked, however, that the results, in absolute terms, are highly dependent on the Manning friction coefficient chosen. For the same return period (5 years) and the same mean sea level (0.5 m), the number of flooded islands increase to five if the Manning coefficient is reduced to 0.025 and decreases to 0 if the Manning coefficient is fixed to 0.2 (see Fig.  S6 to see the number of flooded islands under different Manning coefficients for a mean sea level of 0.5 m). Fig. 4 indicates that the event that occurs once in a 100 years has the potential to flood 24 islands (out of 56) with mean sea levels that are at most 1 m above present-day values. Some of the islands are not expected to suffer wave-induced flooding simply because they are naturally protected being located in sheltered areas of the atolls; this is the case of Vadinolhu and Olhugiri, located in the Kolhumadulu atoll.

### By how much do defenses or islands have to be raised?

We address the second policy question by applying our parameterisation of wave setup and the computation of the wave-induced flooding to estimate by how much the island elevation or its defenses need to be raised in order to cope with mean sea-level rise and waves. The simplest answer given to this question is the *bathtub* approach or *mean SLR allowance* approach, in which island heights or protections are increased by the same amount as mean sea-level rise, neglecting dynamic processes such as wave setup, runup and overtopping that are sensitive to the water depth on the reef that may change with mean sea-level rise. A second answer given in the literature is the one of *total water level hazard allowance*^29^, which consists in raising islands or protections to keep the probability of occurrence of a total water level, defined here as mean sea level plus wave setup, relative to the island elevation constant. This approach accounts for changes in wave setup (that decreases when increasing mean sea level), but not wave run-up and over-topping and hence is not very meaningful when waves are the main driver of flooding. A third, more meaningful approach to adaptation in regions dominated by wave-induced flooding would be *flood hazard allowance*, which consists in raising islands or their protections by an amount that keeps the probability of flooding constant at present-day value.

**Figure 5:**
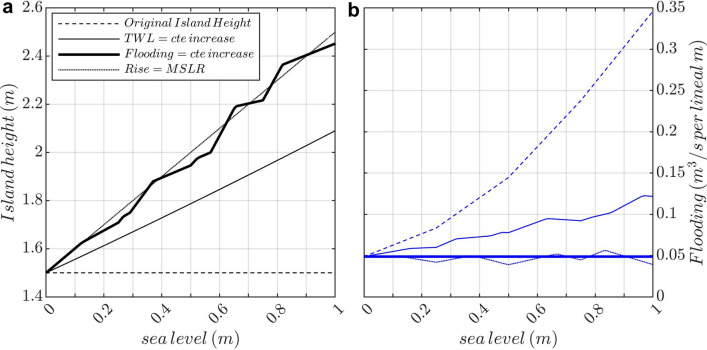
Evolution of the island height (**a**) and flooding (**b**) with projected mean sea level for an archetypal island with 1.5 m of height, 400 m of reef length (Manning coefficient of 0.1) affected by waves with a 4 m of $H_s$ and 20 s of $T_p$ for different approaches of adaptation: no adaptation (keeping the initial island height, dashed lines), increasing the island to keep the total water level (mean sea level + wave setup) constant (thin lines), and increasing the island to keep the flooding constant (thick lines).

For illustration, we apply these three approaches (Fig. 5), together with a no adaptation baseline, to an archetypal island with a height of 1.5 m, 400 m of reef length, a Manning coefficient of 0.1 and affected by wind-waves of 4 m and 20 s of significant wave height and peak period, respectively. Under no adaptation (dashed lines), the flooding increases considerably as mean sea level rises, surpassing the critical flooding threshold of 0.1 m$^3$/s per linear m with a mean sea-level rise of only 31 cm, and reaching a flooding three times larger (from 0.05 to 0.15 m$^3/s$ per lineal m) than its present-day value with a mean sea-level rise of 50 cm. When raising the island a distance equivalent to mean sea-level rise (dotted lines), the associated wave-induced flooding is reduced drastically with respect to the no-adaption option and it remains nearly constant (see “Methods” and Fig. S4). When keeping the total water level relative to island elevation constant (thin solid lines), the amount that the island needs to be raised is smaller than the mean sea-level rise due to the decrease of the wave setup, which is reduced from 1.3 m with 0 m MSLR down to 0.9 m with 1 m MSLR. As a consequence, the wave-induced flooding is larger because maximum water levels do not account for overtopping. It increases by 50% with a 50 cm of mean sea-level rise; note that it does not exceed the critical flooding threshold of 0.1 m$^3$/s per linear m with values of up to 0.85 m of mean sea-level rise. Finally, forcing the flooding to remain unchanged (thick solid lines) translates into an island elevation 20 cm (29 cm) higher than using the approach of keeping the total water level constant for a mean sea-level rise of 50 cm (1 m). The approach of keeping the probability of flooding constant at present-day levels is the most meaningful approach to determine the adaptation needs for reef environments, assuming that the island is protected for the current risks. Interestingly, our results point out that this choice is equivalent to the bathtub approach, in which the island height is raised by the same amount of mean sea-level rise, and considering the flooding due to overtopping is also superior than simply keeping the total water level constant.

Whatever the preferred choice is, the parameterisation of wave setup and the results of the simulations of the wave-induced flooding allow a rapid calculation of these parameters under varying climatic conditions integrating wave impacts, thus supporting coastal adaptation strategies. By specifying a user-defined threshold for wave-induced flooding it is possible to determine under what mean sea-level rise it will be exceeded and, likewise, which is the required allowance to avoid this hazard. The values resulting from the different options, and their consequent impacts, can then be considered by the responsible authorities together with other policy priorities such as investment cycles or budget constraints.

## Discussion

The methodology presented in this study is a useful tool to assess island habitability under mean sea-level rise as a function of morphological characteristics of the island and ocean wave climate. By linking projected mean sea-level rise to their corresponding time horizons under climate change scenarios, we also established a time frame for developing human interventions aimed at keeping (or increasing) the current coastal defenses over the entire Maldivian archipelago as well as for the economic quantification of these actions. The application of the method to other coral reef atolls in different regions is straightforward, simple and fast, allowing to anticipate the concomitant impacts of mean sea-level rise and waves and to assist broad-scale coastal management.

Our work builds upon prior hydrodynamic modelling studies using non-hydrostatic models such as Basilisk or Swash that have demonstrated the capacity to capture wave dynamics in coral reefs^21,30–32^, including validation with field observations^33^. Given a fixed reef morphology and offshore swells, the wave setup during swell events is projected to decrease for higher mean sea-levels according to simulations, which is consistent with observations showing that the wave setup is lower at high tides than at low-tides^34,35^. Recently, Beetham and Kench^10^ used a different numerical scheme to derive a threshold for wave-induced flooding on reef islands and the results from our flooding simulations are coherent with their results (Figure S7). In addition, we have gone a step further by computing the volume of water associated with the flooding, instead of the possibility of occurrence only. The intensity of flooding (the flux of water overtopping the island), as well as the total water level caused by wave setup, are the metrics that are needed for the design of coastal protections. Our new formula for the wave setup is specifically derived for reef islands and will therefore avoid the need to apply modifications of other parameterisations, as done in^36,37^, that were designed for different types of environments, such as beaches^38^.

**Figure 6:**
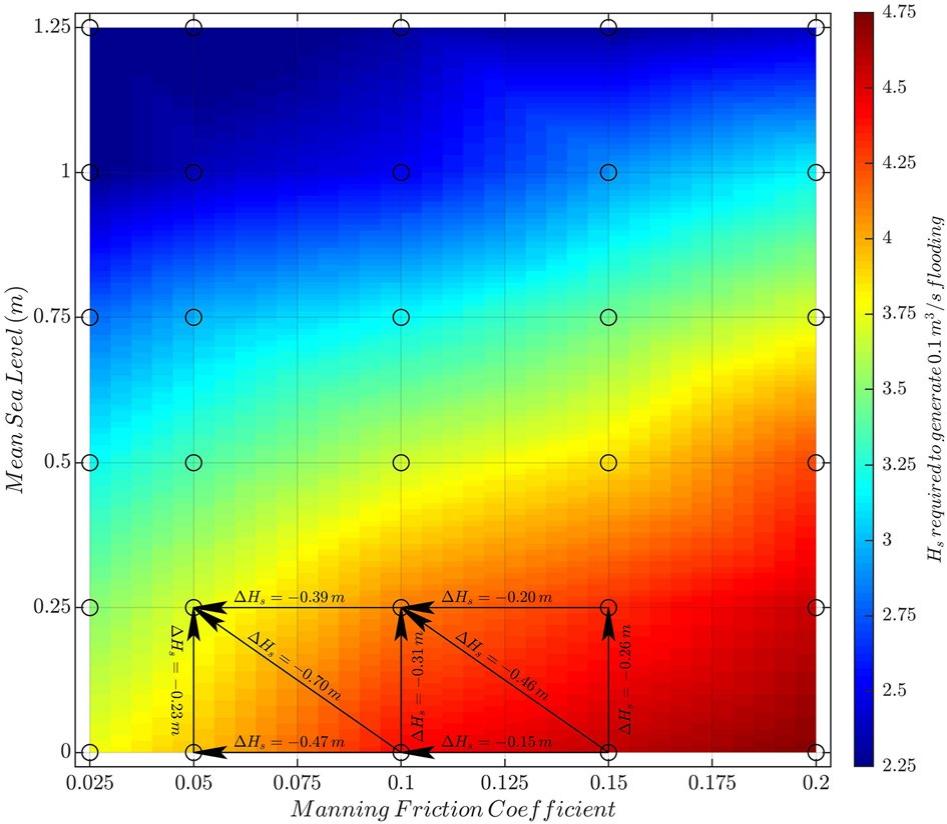
Median significant wave height (median value computed using all reef lengths, island heights and peak periods) required to generate a flooding larger than 0.1 m$^3$/s as a function of Manning friction coefficient (x axis) and mean sea level (y axis). The colourplot is a bi-linear interpolation from the 30 values (black dots) obtained from the numerical simulations. Arrows indicate the $H_s$ change when moving between the two indicated combinations of Manning value and mean sea level.

Coral reef degradation leads to a reduction in the frictional dissipation of waves^39,40^. Figure 6 shows the $H_s$ required in deep waters to generate a flooding larger than 0.1 m$^3$/s as a function of the Manning friction coefficient and mean sea level. The results show that a decrease of the Manning coefficient, linked to degradation of the coral reef, causes a decrease in the $H_s$ needed to generate flooding above the defined threshold. In other words, healthy corals are a more efficient coastal protection. This result is in agreement with^41^ that showed that reef degradation resulted in higher back-reef wave heights (which is translated in higher flooding) for present-day greater than those predicted under projected higher sea levels. From Fig. 6 it is derived that (1) the change in $H_s$ varies less (between 0.23 and 0.31 m) when increasing mean sea level by 0.25 m (projected to occur between 2045 and 2057 depending on the climate scenario, Fig. 3) with constant Manning coefficient rather than when reducing Manning coefficient with constant mean sea level (0.15–0.47 m); (2) the magnitude of the change in $H_s$ strongly depends on the range of Manning values (i.e. the coral reef degradation level): a change of 0.05 in the Manning coefficient could mean a $H_s$ decrease of 0.15 m if the starting Manning value is 0.15 or 0.47 m if the starting value is 0.1 (for 0 m mean sea level); and (3) the largest $H_s$ decrease is found when the coral reef degradation concurs with mean sea-level rise, as both effects add up. Therefore, protecting and restoring coral ecosystems and their structural complexity can mitigate the increasing flooding due to the mean sea-level rise.

Limitations of our study include that we did not distinguished between urban and natural atolls, and, associated to this, that we have not explicitly accounted for either reef accretion in response to mean sea-level rise^42^ or reef degradation due to human interventions or coral mortality due to ocean warming, both of which influence the level of protection provided by reefs^40^. Sediment production and, therefore, topobathymetric changes are nevertheless typically limited in most inhabited islands where human actions are most relevant (see e.g.^2^ for an assessment in the Maldives) although other actions such as over-fishing or pollution can also result in reef degradation in uninhabited islands. In any case, these processes imply changes in the topobathymetric profile that can be accounted for in our parameterisation. Note that as we have modelled a flat island with no vegetation or structures, our results are valid close to the shoreline. More detailed assessments would consider the effects of buildings and defences on water flows, but also the damages of waves on them. Notwithstanding limitations in methodology, our results demonstrate that future atoll flooding in the Maldives will vary significantly depending on the local topobathymetric configuration and swell exposure, thus leaving more or less time for adaptation to take place. Our work is helpful to produce quantitative information for island ranking, depending on the expected impacts and adaptation needs.

## Methods

### Numerical model and simulations

The numerical model used to simulate nearshore wave propagation, from the deep fore reef up to the shoreline, and the flooding was SWASH^22^ (http://swash.sourceforge.net/, version 6.01). It is a phase resolving, non-hydrostatic, open-source numerical model built from the nonlinear shallow water equations that has been extensively tested in a wide range of applications including, but not limited to, wave overtopping on very high cliffs^43^, simulation of the effect of wave frequency spread on wave transformation and swash amplitudes^44^, simulate approximate linear, Stokes, cnoidal and solitary waves^45^, or compute mean wave-driven flow and setup dynamics at a reef-lagoon system^31^ (see http://swash.sourceforge.net/ for a list of studies using SWASH).

A set of 1D numerical simulations along typical coral-reef island profiles (Fig. 1) were performed with different combinations of island height (1 m, 1.25 m, 1.5 m, 1.75 m, 2 m, and 2.5 m) and reef length (from 100 to 600 m with a 100 m step). The profile includes a 100 m-long island with a prescribed constant elevation. The fact that the island is completely flat implies that our simulations do not allow partial flooding, scenario that may still require adaptation. The flat island ends in a beach profile^46^ with a grain size ($D_{50}$) of 1 mm^47,48^ from the top of the island down to 1 m depth. The reef flat is modelled with a 1 m constant depth until reaching the reef-crest. From the limit of the reef-crest down to 60 m depth, the profile follows a combination of bathymetric profiles from two islands of Maldives (Hoarafushi and Madaveli) measured during a field trip on February 2018. Bathymetric measurements have been smoothed to develop an idealized profile. In order to allow waves to fully develop before reaching the reef slope, the 1D domains include a 600m-long flat region at 60 m depth (not shown in Fig. 1). The horizontal resolution of the profiles is 1m and two vertical layers were used if the peak period of the incoming waves was smaller than 15 s (otherwise a single vertical layer was used). Due to the coarse resolution in the vertical, the breaking of the waves was introduced in SWASH with the default parameters ($\alpha =0.6$ and $\beta =0.3$).

60 different combinations of significant wave height ($H_s$) and peak period ($T_p$) were tested, ranging from 0.7 m up to 5.5 m and 5.8 s up to 21.3 s, respectively. These combinations of $H_s$ and $T_p$ were chosen based on the most common values given in the intertropical area ($\pm 25^{\circ }$ latitude, region where most of the coral reef islands are found) of the CFSR hindcast CAWCR Global wind-wave data set^49^ (Figure S1). The wave forcing was generated with a JONSWAP spectrum with $\gamma = 3.3$. The mean sea levels tested ranged from 0 m up to 1.25 m with a 0.25 m step. Changes in mean sea level can also be interpreted as changes in the depth of the reef flat. Tides were not explicitly included but any mean sea level introduced in the simulations, following the same approach as^9^, can be interpreted as a combination of tides plus a permanent mean sea-level rise. For example, 1 m of mean sea level in the simulations can be interpreted as 1 m of permanent mean sea level plus no tidal amplitude or 0.5 m of permanent mean sea level plus 0.5 m of tidal amplitude; thus the same simulation can refer to different time horizons with different tidal amplitude. In order to take into account the inherent stochasticity of generating the incident waves from a JONSWAP spectra, a total of three different simulations with different seed for the random number generator were run for each parameter combination.

The Manning roughness coefficient is one of the most difficult parameters to fix^50^ and even more in 1D simulations since^51^ found that they had to increase the friction coefficient considerably in 1D simulations to match the results of their more realistic 2D simulations. We defined different Manning coefficients for the beach/reef part of the profiles, following^31^. In our case it is considered constant (0.025) over the part of the profile corresponding to island and beach (thin black line on the topobathymetric profile in Fig. 1) and it is changed among simulations over the reef flat and the rest of the profile (thick black line in Fig. 1). The values of the Manning coefficient reported in the literature for coral reefs vary from 0.01 to 0.2 (0.01 in^22^, 0.05 in^52^, 0.1 in^53^ and spatially-varying values of 0.02 (deep ocean), 0.19 (reef flat), and 0.2 (dry land) in^54^). For that reason we tested five different values of the Manning coefficient corresponding to 0.025, 0.05, 0.1, 0.15 and 0.2. With the variations of the six parameters (significant wave height, peak period, mean sea level, island height, reef length and Manning’s coefficient) and the three simulations for each combination of parameters, it makes a total of more than 194,000 numerical simulations.

The simulation period was set to 60 min, with an initial integration time of 0.05 s. The discharge per unit width was saved every 0.2 s at the first point of the simulation domain and later used to compute the wave-induced flooding, as defined above in Eq. (1). The wave setup profile (see an example in Fig. 1) was computed by the model during the last 50 min of simulation to allow a full development of the wave field during the first 10 min.

Performing 1D simulations instead of 2D simulations may result in an overestimation of the wave setup (and consequently of the wave-induced flooding) since the water cannot drain sideways and it accumulates higher against the shoreline. Unfortunately, performing a complete set of 2D simulations is computationally too expensive (running the set of 1D simulations already took more than two months) and it would require the definition of an island shape that would not be representative of all islands. No wave setup or flooding data was available to validate the model results.

### Analyses of the model outputs

For every combination of wave parameters, $H_s$ and $T_p$, the wave setup was determined as the median of the wave setup values of the three corresponding random runs at the grid point where the mean sea level meets the beach profile (see Fig. 1). The flooding, *F*, for each individual simulation was computed as the mean of the discharge per unit width, *Q*, saved at the initial point of the profile during the simulation period, *T*:

1 $$\begin{aligned} F=\frac{1}{T}\int _{0}^{T}Q(t)dt \end{aligned}$$

As for the wave setup, the flooding value for a given ($H_s$,$T_p$) was determined as the median of the flooding values of the three equivalent numerical simulations.

The numerical simulations for which mean sea level was higher than the island height were not considered.

### Parameterisation of wave setup

The dependence between modelled wave setup at the shoreline and each of the six parameters that characterise wave forcing and island morphology is shown in Figure S2. Note that $H_s$ and $T_p$ were combined into a single parameter (Figure S2a). Results in every panel are displayed using boxplots that were calculated fixing one parameter and allowing the others to vary within their prescribed ranges. There is a clear linear relationship ($R^2$ over 0.99) between the wave setup and the product of $H_s$ and $T_p$ (Fig. S2a), irrespective of the values of the other parameters. Likewise, wave setup is reduced with increasing mean sea level (Fig. S2b). The dependence in this case is parameterised as a hyperbole, given that it is bounded by the value at zero mean sea level, with a relationship $a/(b+sea\, level) + c$, leading to a final $R^2$ of 0.99. No dependence was found between wave setup and island morphology (reef length and island height as shown in Fig. S2c,d, respectively) or Manning friction coefficient (Fig. S2e).

Based on the modelled relationships between wave setup and each individual parameter determined above and given that the offshore waves are independent of mean sea level, the following parameterisation was defined:

2 $$\begin{aligned} Wave\,Setup=[a_{HT}\cdot H_s \cdot T_p + b_{HT}]\cdot \left[ \frac{a_{SL}}{b_{SL}+sea\,level} + c_{SL}\right] \end{aligned}$$

The values of the constants were determined using a non-linear-least-squares fit with the $H_s\cdot T_p$ and mean sea level as independent variables and the wave setup as dependent variable, and resulted in:

3 $$\begin{aligned} \begin{aligned} a_{HT}&=0.8425\,s^{-1} \quad b_{HT}=-4.8708\,m \\ a_{SL}&=0.9844\,m^2 \quad b_{SL}=11.7627\,m\quad c_{SL}=-0.0632\,m \end{aligned} \end{aligned}$$

We investigated the robustness of the values of the coefficients through a sensitivity test in which each parameter ($a_{HT}$, $b_{HT}$, $a_{SL}$, $b_{SL}$ and $c_{SL}$) was changed by a random amount, either positive or negative, up to 5% of its adjusted value. Then, we computed the slope, the RMSD and the $R^2$ between the parameterised values in Eq. (2) with the modified coefficients and the simulated (i.e. true) values. The process was repeated 10,000 times for each single coefficient and for all coefficients at once. The results (Fig. S3) point at the constants linked to mean sea-level changes ($a_{SL}$, $b_{SL}$ and $c_{SL}$) as the most sensitive; changes in these constants induced a maximum variation of the slope between 0.75 and 0.97 and an increase in RMSD up to 0.15 m (there are not significant variations of the $R^2$, with values always larger than 0.945). Interestingly, a 5% variation of the parameters corresponding to the ocean waves features ($a_{HT}$ and $b_{HT}$) does not introduce significant changes in the slope, RMSD or $R^2$ between the parameterised wave setup values and the modelled ones.

Other predictive models for wave setup in coral reef islands, such as the Bayesian network model by^55^ (that also provides runup and overtopping), already exist. However, as far as we know, our wave setup parameterisation is the simplest available way to estimate the wave setup in these islands that will avoid the use of adaptations of other wave setup parameterisations (such as^38^) created for other environments different that coral reef islands.

### Computation of flooding

In each numerical run, the flooding was computed as the mean discharge per lineal meter of coastline during one hour of simulation (Eq. 1) measured at the first point of the profile (Fig. 1). The flooding has a dependency with each one of the six parameters analysed, namely $H_s$, $T_p$, reef length, island height, mean sea level and Manning friction coefficient. Consequently, it has been found that the coefficients of an eventual parameterisation are too sensitive to changes in the input values and, therefore, the approach followed for the wave setup has been discarded. Instead, the results of the simulations have been directly applied to compute changes in the flooding with mean sea-level rise. To do so, we first select the simulations with prescribed $H_s$ and $T_p$ (that correspond to a given return period for a given island), reef length and Manning coefficient. An example of the results is illustrated in Fig. S4 for the particular case $H_s=4m$ and $T_p=20s$, a reef length of 500 m and a Manning friction coefficient of 0.1. This selection leaves 36 different simulations for all the possible mean sea levels and island heights simulated (black dots in Fig. S4). The values of the flooding resulting from these 36 simulations are interpolated bi-linearly for mean sea level between 0 m and 1.25 m and for island heights between 1 m and 2.5 m (colour plot in Fig. S4). This so-called flooding map is used in an example with an island with a height of 1.65 m (thick black line in Fig. S4). If the island height remains unchanged as mean sea level rises, the flooding experienced in this particular island is given by the (coloured) values along that same line. Alternatively, if action is taken to require a constant flooding value as in present-day (i.e. as with zero mean sea-level rise), the island height should be raised by the amount indicated with the dashed black line, that corresponds to the isoline of constant flooding. The flooding map also provides information on the value of mean sea level for which the island will suffer a flooding above given threshold; the response to this question is found at the intersection between the line of the island height (thick black line) and the isoline of the given flooding threshold (think black line for 0.1 m$^3$/s). To allow the flooding analysis results to be reproducible, the results of the simulations are available at https://doi.org/10.5281/zenodo.5521394.

### Data from the Maldives

We illustrate the application of the flooding simulations to selected islands in the Maldivian archipelago. To do so, we extracted available information from the morphology of the islands. Firstly, island elevations were obtained from the webpage of the Maldives Land and Survey Authority (http://www.mlsa.gov.mv/psm.php). This organization places permanent survey benchmarks (usually three of them) over islands distributed along the entire archipelago. The benchmarks are not necessarily deployed on the highest point of the island, which means that they could be underestimating the elevation. To minimize this effect to the extent of possible, the elevations were selected as the maximum value registered at the permanent survey benchmarks. Secondly, for every island where there are elevation data, the reef length was determined as the distance between the coastline of the island and the reef crest as measured using satellite images from Google Earth. The direction for which the reef length was measured was defined on the basis of the incoming waves for every island. Finally, the incoming waves were obtained from^9^, where the authors provide maps of the 10, 20, 50, 100, 500, and 1000-year return periods for the $H_s$, $T_p$ and peak direction for the four main directions that reach the Maldives. Using this dataset, we extracted the wave parameters nearby every island with morphological information, obtaining a return level curve of $H_s$ in every island. We adjusted a Generalized Pareto Distribution that allowed interpolating to any arbitrary return period. A total 56 different islands were tested, after discarding those with reef length longer than 700 m (because that was the maximum value in the computed numerical simulations) and those exposed to wave height smaller than 1 m.

The 1D profile used in the numerical simulations is representative of a platform reef island typical of the Maldivian atolls. In addition, we have compared the section of the profile corresponding to the beach (and defined following^46^, see above) with the only available LiDAR high-resolution topo-bathymetry data corresponding to Mahibadhoo island, located at eastern side of the Ari atoll. The comparison (not shown) resulted in an excellent agreement when a grain size $D_{50}$ of 1 mm is used.

### Mean sea-level rise

We use the mean sea-level reconstructions since 1950 of^56^, which combine altimetric or ocean models (Drakkar, Soda) spatial patterns with continuous tide gauge measurements to reproduce regional past mean sea-level changes since 1950, and which have been extensively evaluated^57^. For projected mean sea level in the 21st century we use the AR6 mean sea-level projections in the Maldives^28^ (data available at: https://sealevel.nasa.gov/data_tools/)^17^. We also compute regional mean sea-level projections for the Maldives aligned with the assumption of the Special Report on the Ocean and Crysosphere in a Changing Climate (SROCC^18^), using the same approach as in^58^. AR6 and SROCC projections are similar for all tested scenarios. Finally, we provide a low-confidence, high-end scenario available in the AR6 projections.

## Supplementary Information

Supplementary Information.

## References

[1] Nicholls RJ, Cazenave A (2010). Sea-level rise and its impact on coastal zones. Science.

[2] Duvat VKE (2019). A global assessment of atoll island planform changes over the past decades. WIREs Clim. Change.

[3] Storlazzi CD (2018). Most atolls will be uninhabitable by the mid-21st century because of sea-level rise exacerbating wave-driven flooding. Sci. Adv..

[4] Kench PS, Ford MR, Owen SD (2018). Patterns of island change and persistence offer alternate adaptation pathways for atoll nations. Nat. Commun..

[5] Cazenave A, Cozannet GL (2014). Sea level rise and its coastal impacts. Earth’s Future.

[6] Pedreros R (2018). Relative contribution of wave setup to the storm surge: Observations and modeling based analysis in open and protected environments (Truc Vert beach and Tubuai island). J. Coastal Res..

[7] Wadey M, Brown S, Nicholls RJ, Haigh I (2017). Coastal flooding in the Maldives: An assessment of historic events and their implications. Nat. Hazards.

[8] Hoeke RK (2013). Widespread inundation of pacific islands triggered by distant-source wind-waves. Glob. Planet. Change.

[9] Amores A (2021). Coastal flooding in the Maldives induced by mean sea-level rise and wind-waves: From global to local coastal modelling. Front. Mar. Sci..

[10] Beetham E, Kench PS (2018). Predicting wave overtopping thresholds on coral reef-island shorelines with future sea-level rise. Nat. Commun..

[11] Hinkel J (2019). Meeting user needs for sea level rise information: A decision analysis perspective. Earth’s Future.

[12] Brown S (2020). Land raising as a solution to sea-level rise: An analysis of coastal flooding on an artificial island in the Maldives. J. Flood Risk Manage..

[13] Esteban M (2019). Adaptation to sea level rise on low coral islands: Lessons from recent events. Ocean Coastal Manage..

[14] Masselink G, Beetham E, Kench P (2020). Coral reef islands can accrete vertically in response to sea level rise. Sci. Adv..

[15] Duvat VKE (2021). Risks to future atoll habitability from climate-driven environmental changes. WIREs Clim. Change.

[16] Kane HH, Fletcher CH (2020). Rethinking reef island stability in relation to anthropogenic sea level rise. Earth’s Future.

[17] Magnan A, Duvat V (2019). Towards adaptation pathways for atoll islands insights from the Maldives. Region. Environ. Change.

[18] Oppenheimer, M. *et al.* Sea level rise and implications for low-lying islands, coasts and communities. *in: IPCC Special Report on the Ocean and Cryosphere in a Changing Climate, In press* (2019).

[19] Gussmann, G. & Hinkel, J. Effectiveness of formal and informal coastal governance structures in the maldives: A case of neopatrimonialism. *under review in Global Environmental Change* (2020).

[20] Beetham E, Kench PS, Popinet S (2017). Future reef growth can mitigate physical impacts of sea-level rise on atoll islands. Earth’s Future.

[21] Beetham E, Kench PS, O’Callaghan J, Popinet S (2016). Wave transformation and shoreline water level on Funafuti atoll, Tuvalu. J. Geophys. Res. Oceans.

[22] Zijlema M, Stelling G, Smit P (2011). Swash: An operational public domain code for simulating wave fields and rapidly varied flows in coastal waters. Coast. Eng..

[23] Gourlay M (1996). Wave set-up on coral reefs 2 set-up on reefs with various profiles. Coast. Eng..

[24] Gourlay M (1996). Wave set-up on coral reefs 1 set-up and wave-generated flow on an idealised two dimensional horizontal reef. Coast. Eng..

[25] Woodworth PL (2005). Have there been large recent sea level changes in the Maldive islands?. Glob. Planet. Change.

[26] Gussmann G, Hinkel J (2021). A framework for assessing the potential effectiveness of adaptation policies: Coastal risks and sea-level rise in the Maldives. Environ. Sci. Policy.

[27] Government of the Maldives and United Nations (2007). Joint rapid assessment report on sea swell affected areas. Natl. Disaster Manag. Centre.

[28] Fox-Kemper, B. *et al.* Ocean, cryosphere and sea level change. *In: Climate Change 2021: The Physical Science Basis. Contribution of Working Group I to the Sixth Assessment Report of the IntergovernmentalPanel on Climate Change* (2021). https://www.ipcc.ch/report/ar6/wg1/downloads/report/IPCC_AR6_WGI_Chapter_09.pdf.

[29] Hunter J, Church J, White N, Zhang X (2013). Towards a global regionally varying allowance for sea-level rise. Ocean Eng..

[30] Beetham E, Kench PS, Popinet S (2018). Model skill and sensitivity for simulating wave processes on coral reefs using a shock-capturing Green-Naghdi Solver. J. Coastal Res..

[31] Rijnsdorp DP (2021). A numerical study of wave-driven mean flows and setup dynamics at a coral reef-lagoon system. J. Geophys. Res. Oceans.

[32] Zijlema, M. Modelling wave transformation across a fringing reef using swash. In *ICCE 2012: Proceedings of the 33rd International Conference on Coastal Engineering, Santander, Spain, 1-6 July 2012* (Coastal Engineering Research Council, 2012).

[33] Quataert E, Storlazzi C, van Dongeren A, McCall R (2020). The importance of explicitly modelling sea-swell waves for runup on reef-lined coasts. Coast. Eng..

[34] Vetter EW, Smith CR, de Leo FC (2010). Hawaiian hotspots: enhanced megafaunal abundance and diversity in submarine canyons on the oceanic islands of Hawaii. Mar. Ecol..

[35] Becker JM, Merrifield MA, Ford M (2014). Water level effects on breaking wave setup for pacific island fringing reefs. J. Geophys. Res. Oceans.

[36] Merrifield MA, Becker JM, Ford M, Yao Y (2014). Observations and estimates of wave-driven water level extremes at the marshall islands. Geophys. Res. Lett..

[37] Beck MW (2018). The global flood protection savings provided by coral reefs. Nat. Commun..

[38] Stockdon HF, Holman RA, Howd PA, Sallenger AH (2006). Empirical parameterization of setup, swash, and runup. Coast. Eng..

[39] Quataert E, Storlazzi C, van Rooijen A, Cheriton O, van Dongeren A (2015). The influence of coral reefs and climate change on wave-driven flooding of tropical coastlines. Geophys. Res. Lett..

[40] Sheppard C, Dixon DJ, Gourlay M, Sheppard A, Payet R (2005). Coral mortality increases wave energy reaching shores protected by reef flats: Examples from the Seychelles. Estuarine Coast. Shelf Sci..

[41] Harris DL (2018). Coral reef structural complexity provides important coastal protection from waves under rising sea levels. Sci. Adv..

[42] Woodroffe CD, Murray-Wallace CV (2012). Sea-level rise and coastal change: The past as a guide to the future. Quatern. Sci. Rev..

[43] Amores A, Marcos M, Carrió DS, Gómez-Pujol L (2020). Coastal impacts of storm Gloria (January 2020) over the northwestern mediterranean. Nat. Hazards Earth Syst. Sci. Discus..

[44] Ruju A, Lara JL, Losada IJ (2019). Numerical assessment of infragravity swash response to offshore wave frequency spread variability. J. Geophys. Res. Oceans.

[45] Ruffini G, Heller V, Briganti R (2019). Numerical modelling of landslide-tsunami propagation in a wide range of idealised water body geometries. Coast. Eng..

[46] Dean, R. G. Equilibrium beach profiles: Characteristics and applications. *Journal of Coastal Research* **7**, 53–84 (1991). http://www.jstor.org/stable/4297805.

[47] Perry CT, Morgan KM, Salter MA (2016). Sediment generation by Halimeda on atoll interior coral reefs of the southern Maldives: A census-based approach for estimating carbonate production by calcareous green algae. Sed. Geol..

[48] East HK, Perry CT, Beetham EP, Kench PS, Liang Y (2020). Modelling reef hydrodynamics and sediment mobility under sea level rise in atoll reef island systems. Glob. Planet. Change.

[49] Hemer MA, Trenham CE, Durrant T, Greenslade D (2015). Cawcr Global Wind-Wave 21st Century Climate Projections (v1). Data Collection.

[50] Rosman JH, Hench JL (2011). A framework for understanding drag parameterizations for coral reefs. J. Geophys. Res. Oceans.

[51] Van Dongeren A (2013). Numerical modeling of low-frequency wave dynamics over a fringing coral reef. Coast. Eng..

[52] Prager EJ (1991). Numerical simulation of circulation in a caribbean-type backreef lagoon. Coral Reefs.

[53] Kraines S, Yanagi T, Isobe M, Komiyama H (1998). Wind-wave driven circulation on the coral reef at bora bay, miyako island. Coral Reefs.

[54] Cialone, M. A. & Smith, J. M. Wave transformation modeling with bottom friction applied to southeast oahu reefs. In *10th International workshop on Wave Hindcasting and Forecasting & Coastal Hazard Assessment*, 1–12 (Citeseer, 2007).

[55] Pearson SG, Storlazzi CD, van Dongeren AR, Tissier MFS, Reniers AJHM (2017). A bayesian-based system to assess wave-driven flooding hazards on coral reef-lined coasts. J. Geophys. Res. Oceans.

[56] Meyssignac B, Becker M, Llovel W, Cazenave A (2012). An assessment of two-dimensional past sea level reconstructions over 1950–2009 based on tide-gauge data and different input sea level grids. Surv. Geophys..

[57] Carson M (2017). Regional sea level variability and trends, 1960–2007: A comparison of sea level reconstructions and ocean syntheses. J. Geophys. Res. Oceans.

[58] Thiéblemont R, Le Cozannet G, Toimil A, Meyssignac B, Losada IJ (2019). Likely and high-end impacts of regional sea-level rise on the shoreline change of European sandy coasts under a high greenhouse gas emissions scenario. Water.

[59] Slangen ABA (2014). Projecting twenty-first century regional sea-level changes. Clim. Change.

