# Author Correction: Coastal flooding and mean sea-level rise allowances in atoll island

**DOI:** 10.1038/s41598-022-06548-2

**Published:** 2022-02-09

**Authors:** Angel Amores, Marta Marcos, Gonéri Le Cozannet, Jochen Hinkel

**Affiliations:** 1 Instituto Mediterráneo de Estudios Avanzados (UIB-CSIC), Esporles, Spain; 2 Departament de Física (UIB), Palma, Spain; 3 French Geological Survey (BRGM), Orléans, France; 4 Global Climate Forum (GCF), Berlin, Germany

Correction to: *Scientific Reports* 10.1038/s41598-022-05329-1, published online 14 January 2022

The original version of this Article contained an error in the Acknowledgements section.

“This study was supported by the FEDER/Ministerio de Ciencia, Innovación y Universidades – Agencia Estatal de Investigación through the MOCCA project (grant no. RTI2018-093941-B-C31) and by the INSeaPTION Project that is part of ERA4CS, an ERANET initiated by JPI Climate, and funded by Ministerio de Economía, Industria y Competitividad-Agencia Estatal de Investigación (ES) (Grant number PCIN-2017-038), BMBF (DE), NOW (NL) and ANR (FR) with co-funding by the European Union (Grant 690462).”

now reads:

“This study was supported by the project RTI2018-093941-B-C31 supported by MCIN/ AEI 10.13039/501100011033 and by FEDER Una manera de hacer Europa and by the INSeaPTION Project that is part of ERA4CS, an ERANET initiated by JPI Climate, and funded by Ministerio de Economía, Industria y Competitividad-Agencia Estatal de Investigación (ES) (Grant number PCIN-2017-038), BMBF (DE), NOW (NL) and ANR (FR) with co-funding by the European Union (Grant 690462).”

The original Article has been corrected.

