# Supplementary material for: Coastal flooding and mean sea-level rise allowances in atoll island
Source: Sci Rep. 2022 Jan 24;12:1281. doi: 10.1038/s41598-022-05329-1 (PMC8786857; doi:10.1038/s41598-022-05329-1)
Supplement: Supplementary file 1 — Supplementary Information. [file 41598_2022_5329_MOESM1_ESM.pdf]

Coastal flooding and mean sea-level rise allowances in atoll island: Supplementary Material

ANGEL AMORES*[1], MARTA MARCOS[1,2], GONÉRI LE COZANNET[3], AND JOCHEN HINKEL[4]

[1] Instituto Mediterráneo de Estudios Avanzados (UIB-CSIC), Esporles, Spain.
[2] Departament de Física (UIB), Palma, Spain.
[3] French Geological Survey (BRGM), Orléans, France.
[4] Global Climate Forum (GCF), Berlin, Germany.

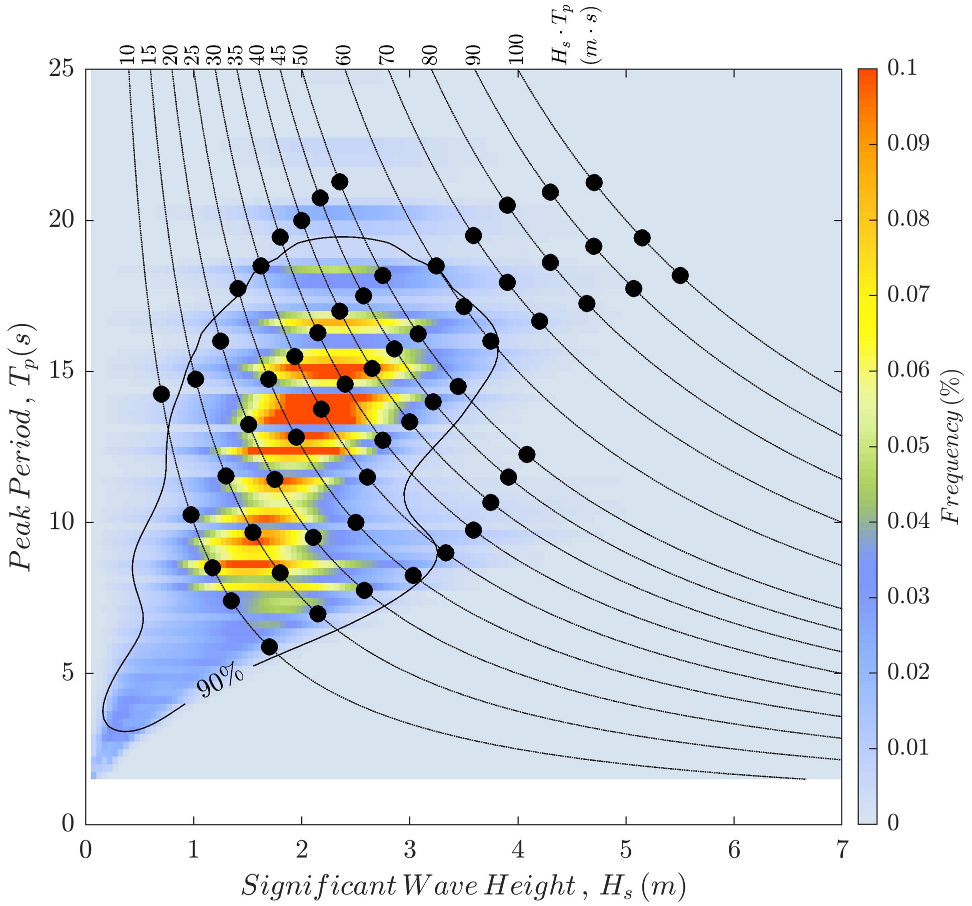

Figure S1. Selection of the combination of H_s and T_p values used in the 1D SWASH numerical simulations. The density plot represents the frequency that a combination of H_s and T_p is given in the intertropical areas (±25° latitude, region where most of the coral reef islands are found) of the CFSR hindcast CAWCR Global wind-wave data set[1]. The black contour represents the area containing the maximum frequency and enclosing 90% of the H_s and T_p combinations. Taking advantage of previous experience, the 60 different combinations used for the simulations (black dots) has been chosen to include the most frequent combination as well as the extremes following lines of constant $H_s \cdot T_p$ (dotted contour lines).

*angel.amores@uib.es

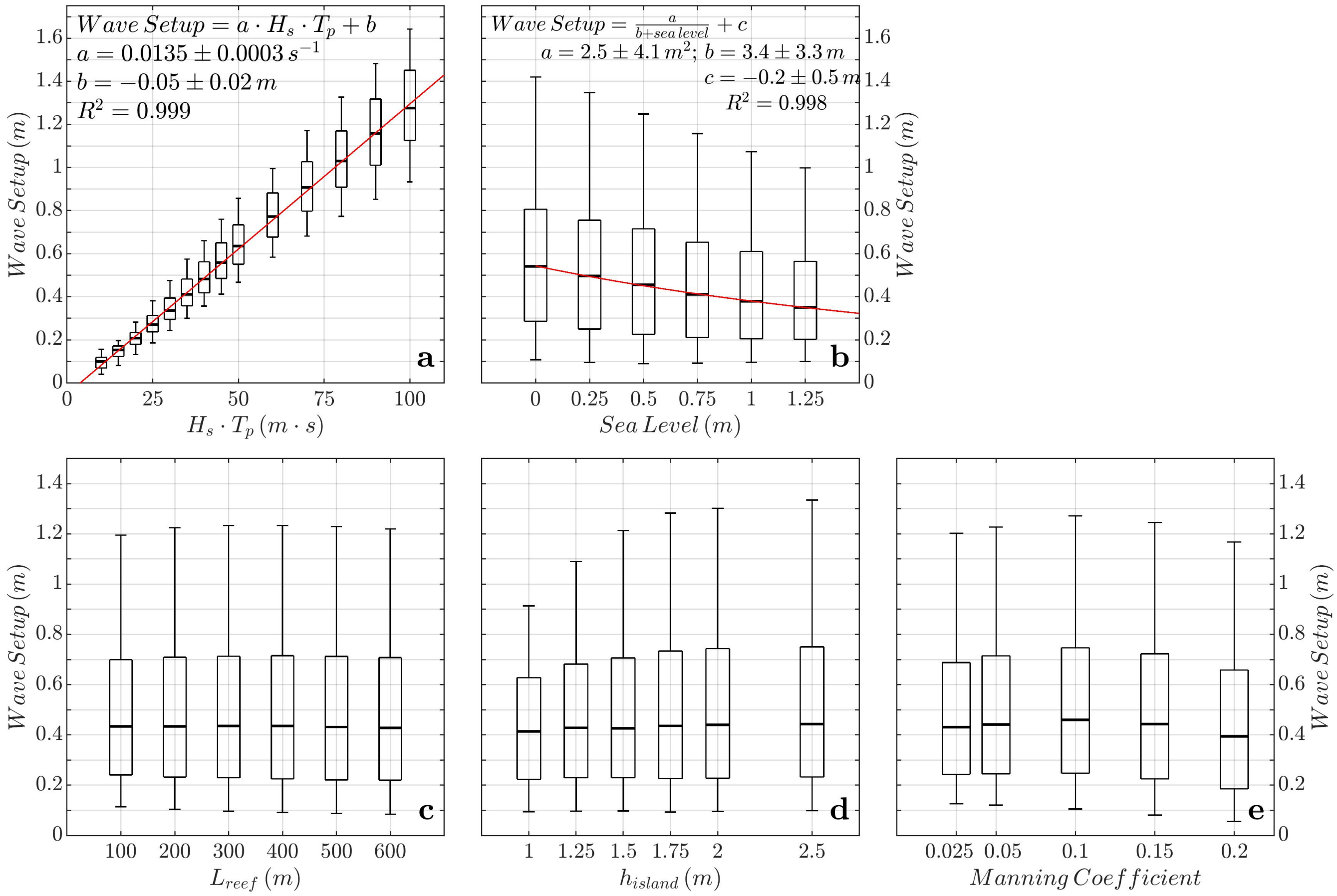

Figure S2. Dependence of the wave setup with: a) significant wave height multiplied by peak period; b) sea level; c) reef length; d) island height; and e) Manning's roughness coefficient. The central value of each box plot represents the median value, the lower (upper) limit of the box is the 25% (75%) quantile and the whiskers show the range of values between the 5% and 95% quantiles.

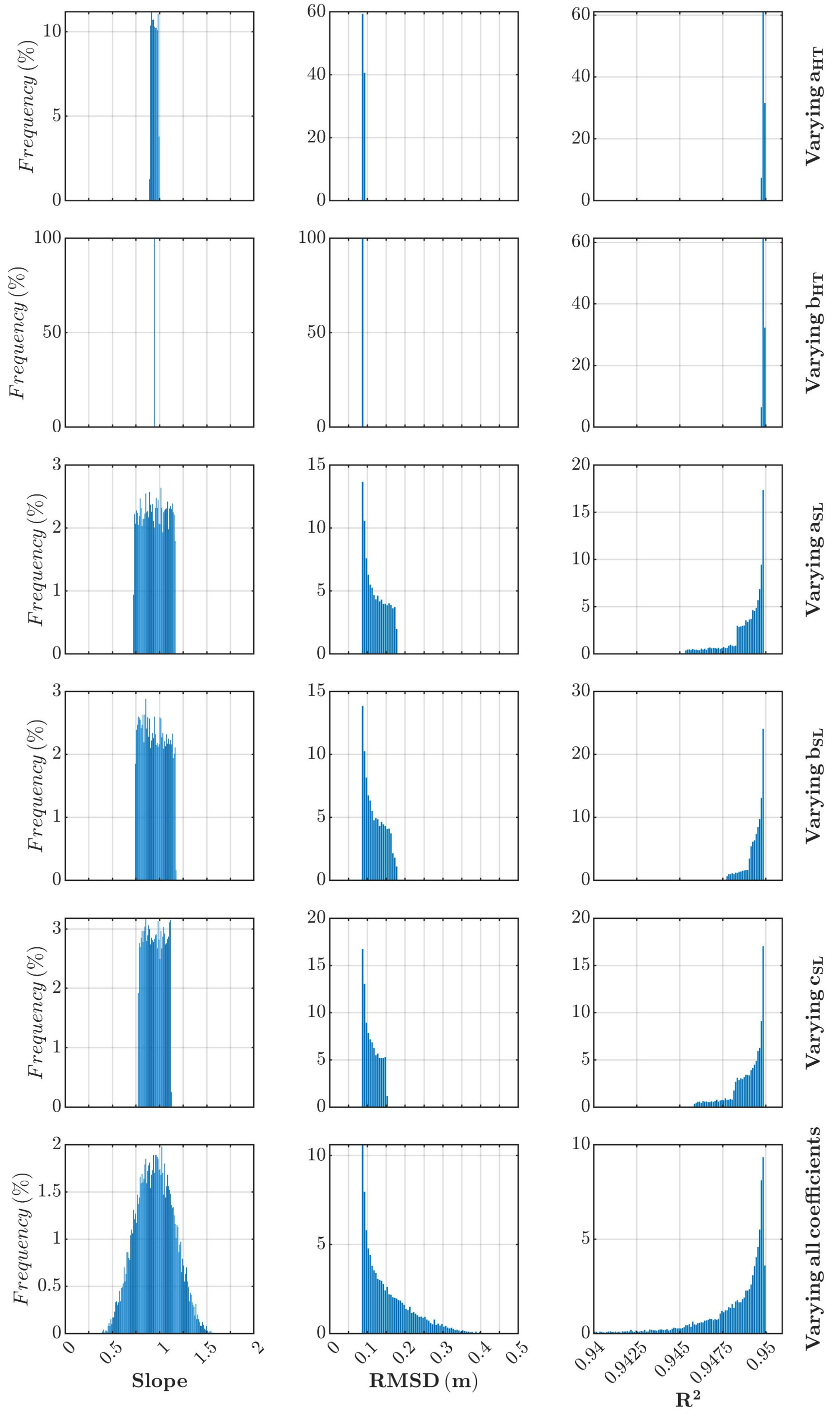

Figure S3. Sensitivity tests for adjusted wave setup parameters in equation 3. Each parameter is randomly changed by up to 5% of its value and the rest are adjusted. Histograms represent the results of the process repeated 10000 times.

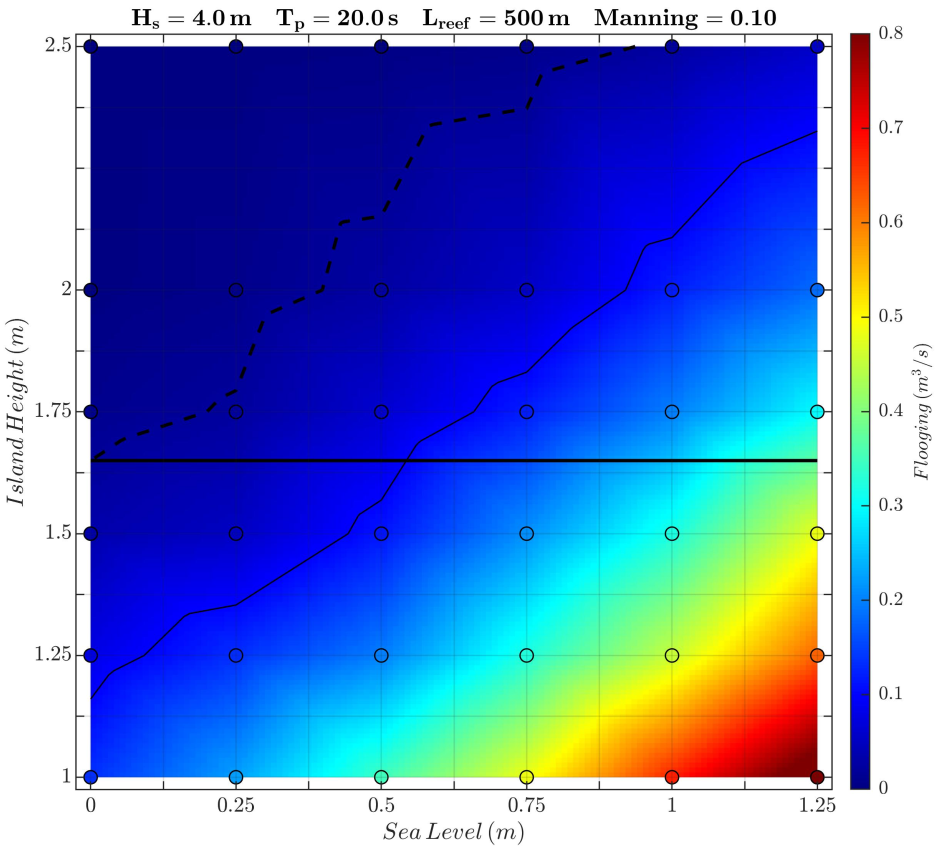

Figure S4. Dependence of the flooding with sea level and island height for incoming waves with $H_s = 4m$ and $T_p = 20s$, a reef length of 500 m and a Manning friction coefficient of 0.1. The colorplot is a bi-linear interpolation from the 36 values (black dots) obtained from the numerical simulations. The horizontal thick black line indicates the flooding that an island with 1.65 m would suffer with sea-level rise is no action is taken. The dashed black line indicates the evolution of the island height with sea-level rise if present-day flooding is required to remain constant. Thin black line indicate the isoline of a used-define flooding threshold (in this case $0.1m^3/s$). The intersection between this line and the horizontal line (that shows the island height) indicates the sea level at which the user defined flooding threshold is over-passed.

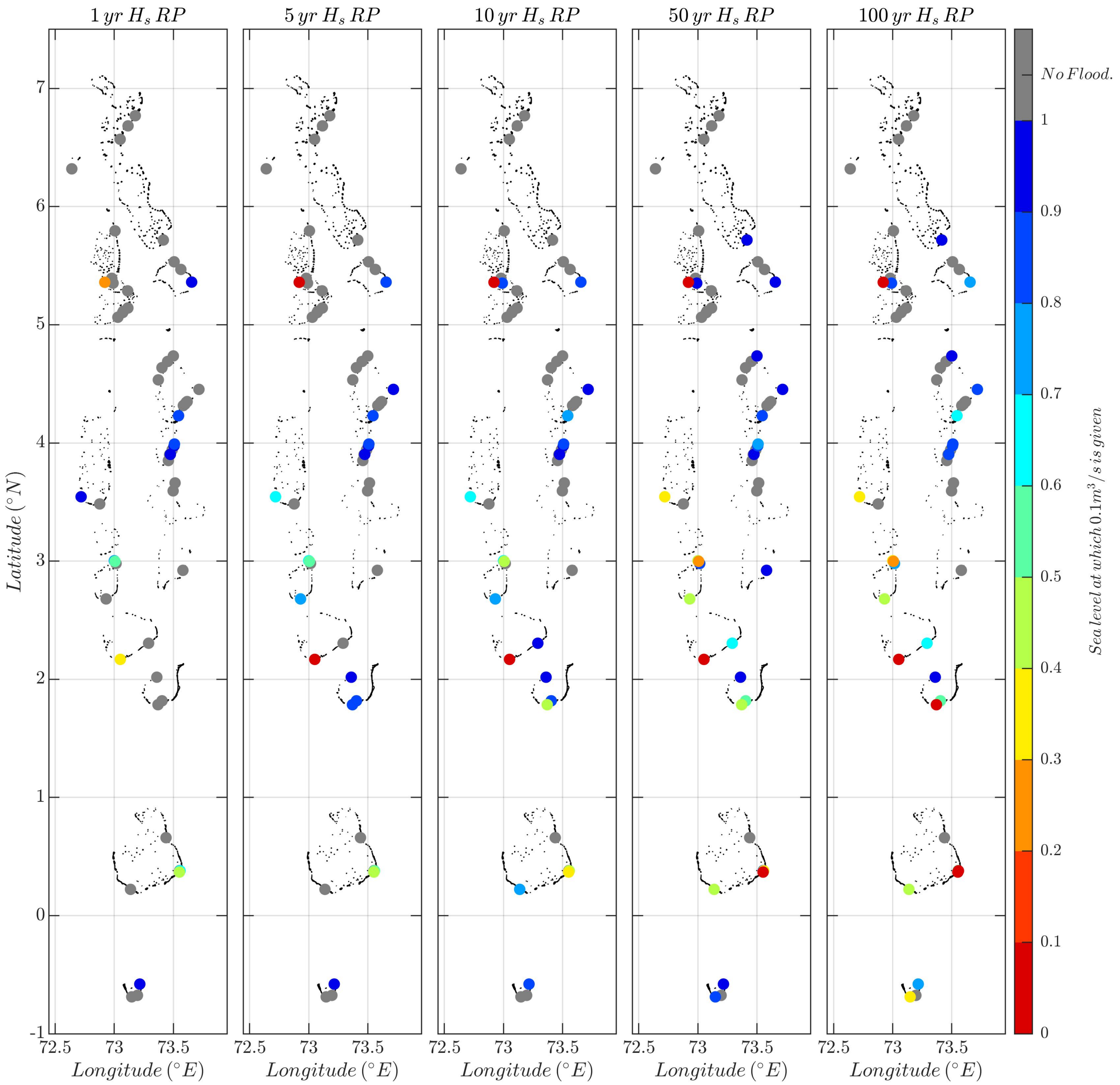

Figure S5. Same as Fig. 4 but with a Manning friction coefficient of 0.05.

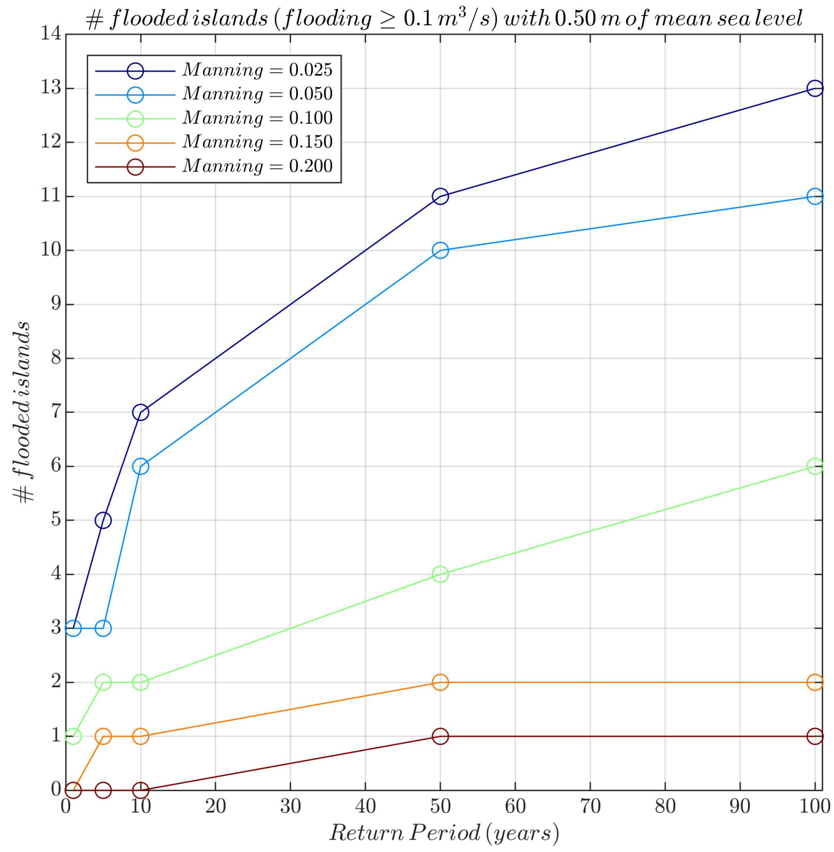

Figure S6. Number of island wave-induced flooded by more than $0.1m^3/s$ with 0.5 m of mean sea level under different return periods and for different Manning friction coefficient. The total number of flooded islands for each return period is highly dependent on the Manning coefficient.

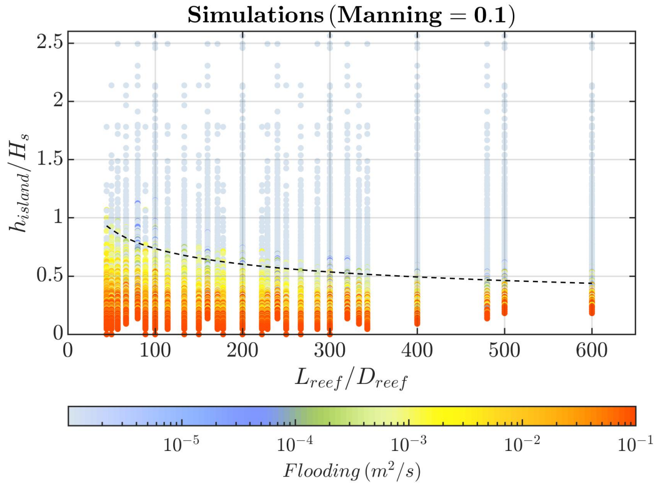

Figure S7. Comparison between the flooding results from our simulations (Manning friction coefficient fixes to 0.1) with the flooding threshold derived by [2] (black dashed line in each panel). Note that we used different mean sea levels while keeping constant to 1 m the reef depth, whereas [2] changed their reef depth (D_{reef}) and kept mean sea level as a constant value. Thus, to perform the comparison we computed the different reef depths as 1 m plus the corresponding mean sea level value. Consequently, our island height is transformed as the original island height minus the sea level to be consistent. Colours (in logarithmic colour scale) indicate the volume of flooding (in m^3/s per linear meter of coastline). Our flooding values above the threshold are always below 0.01 m^2/s.

REFERENCES

1. M. A. Hemer, C. E. Trenham, T. Durrant, and D. Greenslade, “Cawcr global wind-wave 21st century climate projections (v1),” CSIRO. Data Collect. (2015).
2. E. Beetham and P. S. Kench, “Predicting wave overtopping thresholds on coral reef-island shorelines with future sea-level rise,” Nat. Commun. **9**, 3997 (2018).
